# A rapid methods development workflow for high-throughput quantitative proteomic applications

**DOI:** 10.1371/journal.pone.0211582

**Published:** 2019-02-14

**Authors:** Yan Chen, Jonathan Vu, Mitchell G. Thompson, William A. Sharpless, Leanne Jade G. Chan, Jennifer W. Gin, Jay D. Keasling, Paul D. Adams, Christopher J. Petzold

**Affiliations:** 1 Joint BioEnergy Institute, Lawrence Berkeley National Laboratory, Emeryville, CA, United States of America; 2 Biological Systems and Engineering Division, Lawrence Berkeley National Laboratory, Berkeley, CA, United States of America; 3 Department of Plant and Microbial Biology, University of California, Berkeley, CA, United States of America; 4 Department of Bioengineering, University of California Berkeley, Berkeley, CA, United States of America; 5 Department of Chemical and Biomolecular Engineering, University of California, Berkeley, CA, United States of America; 6 The Novo Nordisk Foundation Center for Biosustainability, Technical University of Denmark, Lyngby, Denmark; 7 Molecular Biophysics and Bioimaging, Lawrence Berkeley National Laboratory, Berkeley, CA, United States of America; Pacific Northwest National Laboratory, UNITED STATES

## Abstract

Recent improvements in the speed and sensitivity of liquid chromatography-mass spectrometry systems have driven significant progress toward system-wide characterization of the proteome of many species. These efforts create large proteomic datasets that provide insight into biological processes and identify diagnostic proteins whose abundance changes significantly under different experimental conditions. Yet, these system-wide experiments are typically the starting point for hypothesis-driven, follow-up experiments to elucidate the extent of the phenomenon or the utility of the diagnostic marker, wherein many samples must be analyzed. Transitioning from a few discovery experiments to quantitative analyses on hundreds of samples requires significant resources both to develop sensitive and specific methods as well as analyze them in a high-throughput manner. To aid these efforts, we developed a workflow using data acquired from discovery proteomic experiments, retention time prediction, and standard-flow chromatography to rapidly develop targeted proteomic assays. We demonstrated this workflow by developing MRM assays to quantify proteins of multiple metabolic pathways from multiple microbes under different experimental conditions. With this workflow, one can also target peptides in scheduled/dynamic acquisition methods from a shotgun proteomic dataset downloaded from online repositories, validate with appropriate control samples or standard peptides, and begin analyzing hundreds of samples in only a few minutes.

## Introduction

Reports on the poor reproducibility of scientific results [1] highlight the need for better experimental design, greater effort devoted to validation of novel discoveries, improved hypothesis testing, and stricter publishing requirements. These efforts are especially important for systems-wide studies conducted in laboratories across the world that are commonplace in proteomic research [2–4]. For instance, discovering and validating novel protein biomarkers are key to building clinical diagnostics and development of precision medicine. Moreover, quantifying proteins from microbes contributes to successful comparative analysis of environmental isolates and engineered microbes for production of biofuels and bioproducts. These concerns are driving improvement in analytical protocols, data quality metrics, and reporting [5]. However, to implement appropriately powered, statistically significant studies of biological systems with low signal to noise, many samples must be analyzed, which is a significant challenge even for the most well-resourced proteomic groups [6].

Much of proteome research follows a two-step process: a discovery step to identify proteins of interest followed by subsequent quantitative experiments on a subset of proteins, yet navigating between the discovery and application experiments is a time-consuming process. In part, to optimize this process, data dependent (DDA) and data independent acquisition (DIA) methods have been developed to enable discovery and quantitation in a single data acquisition. This works well for experiments with limited number of samples where comprehensive proteome coverage is needed. However, once targets are identified as potential biomarkers or proteins of interest, efforts switch to validation exercises that involve large quantities of samples, thus demanding high-throughput targeted proteomic assays. Selected reaction monitoring (SRM) targeted proteomic assays provide high sensitivity, dynamic range, specificity, and are amenable to large numbers of samples [7]. Despite recent advances in SRM method development [7], continued development of Skyline [8], and significant steps forward in community standard and guidelines [9–11], utilizing DDA and DIA data to develop SRM methods is a time consuming process. An extreme example of this challenge is the development of the Human SRMAtlas [12] wherein over 166,000 proteotypic peptides were individually chemically synthesized to develop MRM assays for ~20,000 proteins. Naive, *in silico*-derived, SRM transition prediction [7] for proteins of interest generally provides many potential peptides that must be screened to identify the best ones for quantification experiments. Attempts to reduce the number of potential number of candidates by using computational methods (e.g., ESPP [13], PeptidePicker [14], PeptideSieve [15]) or empirical selection peptides based on previous data (e.g., from PRIDE [16], PeptideAtlas [17]) are promising but additional factors such as different experimental conditions, data acquisition methods, variable retention times, and low abundance of the proteins of interest often limit the successful application of these methods. Community resources such as SRMAtlas [12], PRIDE [16], Panorama [18], or the BioDiversity Library [19,20], a collection of proteomic data comprising of over 100 bacterial and archaeal organisms, as well as commercial software tools such as Spectrum Mill and the newly released SpectroDive have been built to overcome these challenges, yet significant methods development is typically still necessary. Likewise, research by Prakash and co-workers showed how spectral libraries are powerful way to select SRM transitions and confirm the identity of peptides in SRM methods [21]. Recently, Schilling et al. [22] developed a workflow to rapidly utilize data acquired via shotgun proteomics to design targeted experiments for accurate and precise quantitation of up to 500 peptides on the same instrument. The success of this workflow pointed to the great potential of using established proteome spectral libraries to develop targeted peptide assays. Yet, transfer of peptide target information, including retention times, between different types of instruments is challenging due to variable chromatography, ionization, sensitivity, and fragmentation conditions resulting in long method development times.

In this work we describe a workflow using data acquired from shotgun proteomic experiments and retention time prediction methods to rapidly develop high-throughput targeted proteomic assays. This workflow simplifies validation of peptides identified from shotgun proteomic experiments and significantly reduces development time of high-throughput quantitative SRM assays. It is enabled by highly reproducible peptide retention times from standard-flow chromatography systems, comprehensive spectral libraries produced from DDA experiments, and tools developed for Skyline, such as the iRT calculator [23]. The workflow is instrument agnostic and performs well by using resources from online proteomic repositories to inform target peptide selection.

## Materials and methods

### Strains and medium

*Escherichia coli* DH 5α, *Saccharomyces cerevisiae* S288C, *Corynebacterium glutamicum*, *Agrobacterium tumefaciens*, and *Rhodosporidium toruloides*, and *Pseudomonas putida* KT2440 strains were cultured in house for the purpose of constructing a proteome spectral libraries. *E*. *coli* DH 5α was grown overnight in Luria broth (LB) medium at 37°C, shaking at 200 RPM. *S*. *cerevisiae* S288C was grown overnight in YPD medium at 30ºC, shaking at 200 RPM. *P*. *putida* was maintained on LB broth, while proteomics experiments were conducted in modified MOPS minimal media supplemented with 10mM of the indicated carbon source. *P*. *putida* was grown in 25mL of media in 250mL Erlenmeyer flasks at 30°C with 200 RPM shaking. Cells were harvested by centrifugation and the cell pellets were frozen at -80°C until further processing.

### Proteomic sample preparation

Protein extraction from *E*. *coli* and other gram negative organisms was accomplished using a chloroform/methanol precipitation method, previously described [24]. Cell pellets (~10 OD/mL) were resuspended in 400 μl of methanol and briefly vortexed, followed by sequential additions of 100 μl of chloroform and 300 μl of water with short intervals of vortexing in between. For *S*. *cerevisiae* cultures, cell pellets were transferred into a PCR plate, then re-suspended in 60 μl of methanol and 100 μl of chloroform. Approximately 50 μl of Zirconia/Silica beads (0.5 mm diameter; BioSpec Products, Bartlesville, OK) were then added to each well. The plate was then sealed and bead beat for 5 cycles of 1 minute bead beating followed by 30 seconds on ice. The supernatants were transferred into a new plate and 30 μl of water was added to each well. The plate was mixed by pipetting and then centrifuged for 10 minutes at maximum speed to induce the phase separation. The methanol and water layers were removed, then 60 μl of methanol was added to each well. The plate was centrifuged for another 10 minutes at maximum speed, then the supernatant chloroform and methanol layers were decanted. The protein pellet was resuspended in 100 mM ammonium bicarbonate buffer supplemented with 20% v/v methanol, and protein concentration was determined by the DC assay (BioRad, Hercules, CA). Prior to protein trypsin digestion at a concentration of 1 mg/mL, protein reduction was accomplished using 5 mM tris 2-(carboxyethyl)phosphine (TCEP) for 30 min at room temperature, and alkylation was performed with 10 mM iodoacetamide (IAM; final concentration) for 30 min at room temperature in the dark. Overnight digestion with trypsin was accomplished with a 1:50 w:w trypsin:total protein.

### LC-MS analysis

Peptides were eluted into the mass spectrometer via a gradient with initial starting condition of 5% buffer B (0.1% v/v formic acid in acetonitrile) and 95% buffer A (0.1% v/v formic acid in water). For analysis of all shotgun proteomic experiments, buffer B was increased to 35% over 120 min. Buffer B was then increased to 50% over 5 min, then up to 90% over 1 min, and held for 7 min at a flow rate of 0.6 mL/min, followed by a ramp back down to 5% B over 1 min where it was held for 6 min to re-equilibrate the column to original conditions. Peptides were introduced to an Agilent 6550 QToF mass spectrometer from the Agilent 1290 UHPLC by using a Jet Stream source (Agilent Technologies) operating in positive-ion mode (3,500 V). Source parameters employed gas temp (250°C), drying gas (14 L/min), nebulizer (35 psig), sheath gas temp (250°C), sheath gas flow (11 L/min), VCap (3,500 V), fragmentor (180 V), OCT 1 RF Vpp (750 V). The data were acquired with Agilent MassHunter Workstation Software, LC/MS Data Acquisition B.05.00 (Build 5.0.5042.2) operating in Auto MS/MS mode whereby the 20 most intense ions (charge states, 2–5) within 300–1,400 m/z mass range above a threshold of 1,500 counts were selected for MS/MS analysis. MS/MS spectra (100–1,700 m/z) were collected with the quadrupole set to “Medium” resolution and were acquired until 45,000 total counts were collected or for a maximum accumulation time of 333 ms. Former parent ions were excluded for 0.1 min following MS/MS acquisition.

All SRM methods development and assays were performed on an Agilent 6460 QQQ mass spectrometer system coupled with an Agilent 1290 UHPLC system (Agilent Technologies). Unless stated otherwise, same amount peptide biomass as used in shotgun proteomics was separated by a Sigma–Aldrich Ascentis Peptides ES-C18 column (2.1 mm × 50 mm, 2.0 μm particle size, operated at 60°C) at 0.400 mL/min standard flow rate. Peptides were ionized by using an Agilent Jet Stream source (Agilent Technologies) operating in positive-ion mode with the following parameter settings: Sheath Gas flow = 11 L/min, Sheath Gas Temperature = 350°C, Nozzle Voltage = 1000 V, Nebulizing Pressure = 30 psi, Chamber Voltage = 4500 V. To calibrate iRT standards in various chromatographic conditions, a standard method was utilized with 25-ms dwell time per transition and Q1 and Q3 resolution set to Unit.

### Spectral library construction in Skyline

All spectral libraries of the in house and online data repository resources acquired proteome were constructed by using Skyline software version 4.10 (MacCoss Lab Software. https://skyline.ms/project/home/software/Skyline/begin.view) [8]. Briefly, the mass spectrometry raw data was converted to .mgf file either by employing MassHunter Workstation Software, Qualitative Analysis (Version B.07.00 Service Pack 1, Agilent Technologies) or ProteoWizard version 2.1. Resultant data files were searched against the latest Uniprot proteome FASTA files of each organism using Mascot search engine version 2.3.02 (Matrix Science) with a peptide tolerance of ±50 ppm and MS/MS tolerance of ±0.1 Da; fixed modifications Carbamidomethyl (C); variable modifications Oxidation (M); up to one missed cleavage for trypsin; peptide charge 2+, 3+, and 4+; and the instrument type was set to ESI-QUAD-TOF. The search results were loaded and analyzed by Scaffold v4.6.1 (Proteome Software Inc.) with protein and peptide threshold filters set at 1.0% FDR, and minimum peptide detection set at 1. The mzXML files were exported from Scaffold and imported into Skyline via peptide search function. All Skyline files of the results described above are available through Panoramaweb [18] (Short Panoramaweb link: https://panoramaweb.org/rapid-shotgun-to-SRM-workflow.url). LCMS Data generated in this study are available via ProteomeXchange with identifier PXD011212.

### Dynamic SRM methods

For each proteome spectrum library, at least 12 detected peptides were picked as landmark standards across the entire gradient to establish the iRT calculator. The retention time of targeted peptides in various LC parameters were predicted based on the retention time adjustment of reference peptides that were empirically determined by a standard SRM method described above. For comparison of in-house and public data repository spectral libraries the peptides were selected by intensity and overlap between the libraries. For scheduled SRM assay methods, a retention time window of 0.3–0.4 min with a target cycle time of 1 s to yield fewer than 200 concurrent transitions. Protein targets of pathways in *P*. *putida* KT2440 were collected from literature review and databases, such as KEGG and MetaCyc. These proteins were selected from our spectral libraries for dynamic SRM method development via the established workflow. From the protein targets, the top 5 peptides and their top 6 fragment ions based on library pick intensity rank were selected and their retention times were predicted via iRT calculators. A 1.2 min retention time window, fewer than 200 concurrent transitions, and target cycle time of 0.8 s in a 5.5 minutes LC gradient were used for data acquisition except for carbon metabolism pathway proteins in *P*. *putida* KT2440, where a 2 min retention time window and target cycle time of 1.0 s in a 20 minutes LC gradient were used. The summed peptide peak area of the proteins were used for quantitative analysis.

## Results

### MRM method development workflow

**Fig 1:**
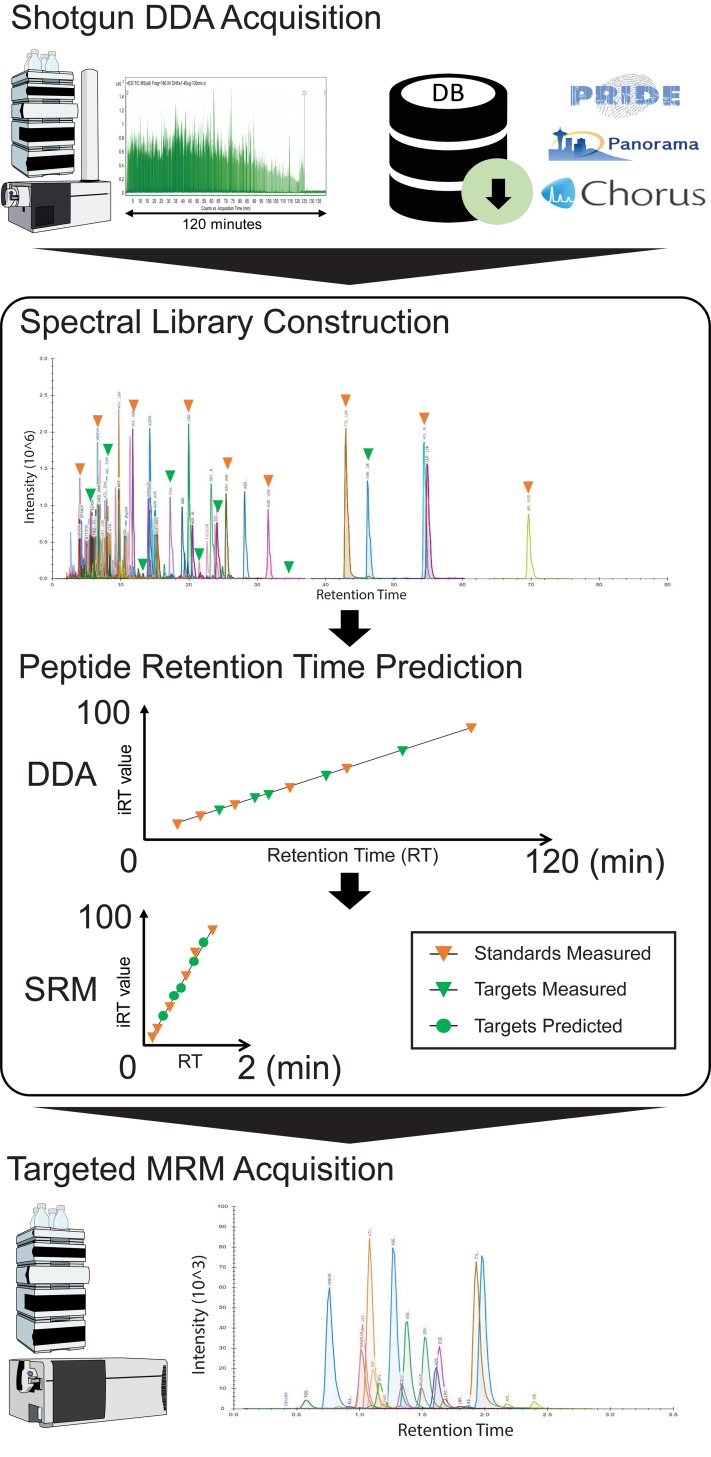
Workflow to rapidly develop SRM targeted proteomic methods from shotgun proteomic data. (a) Identification of proteins from either in-house shotgun DDA acquisition using an LC-QToF or data downloaded from public MS data repositories; (b) construction of a proteome spectral library from raw data containing retention times for a set of host-specific reference peptides and peptides of interest; (c) prediction of targeted peptide retention time based on the spectral library and measured retention times of reference peptides in a new chromatography gradient; (d) predicted RT of targeted peptide used without further methods optimization in a rapid SRM method.

To reduce the amount of time necessary to develop targeted proteomic methods we established a workflow to utilize the peptide information acquired from shotgun proteomic experiments. This eliminates (or greatly reduces) the need for *in silico* selected reaction monitoring (SRM) design or extensive method transfer experiments. The workflow involves the following steps (summarized in Fig 1): (a) acquire data-dependent acquisition (DDA) data or download data from online repositories (b) Construct a proteome spectral library from shotgun proteomics data; (c) Select reference peptides and apply retention time calculator to predict peptide retention times for scheduled SRM methods that use short chromatography gradients; (d) acquire SRM data by using dynamic/scheduled methods.

Spectral libraries are an integral part of DIA and DDA proteomic workflows. They combine data acquisition parameters such as retention time, precursor mass, and product ion masses with the results of peptide searches. They enhance reproducible quantification of peptides that may not have been selected for MS/MS fragmentation, but they are also useful for transferring methods to different instruments or to SRM methods for targeted analyses. Yet, complicating the implementation of the workflow described in Fig 1 are the differences between MS/MS fragmentation processes on various instruments (*e*.*g*., ion-trap (resonance) CID versus QqQ (beam-type) CID processes). Furthermore, in the case of nano-LC instrumentation, the de facto standard for proteomics research, the run-to-run variability of peptide retention times could range from 0.5 to 2.2 minutes depending on the nano-LC platform [25], which would require retention time scanning windows of five minutes or more when modifying the chromatographic conditions [23]. While this is not a major problem for small numbers of peptides it complicates attempts to target large numbers of peptides in SRM methods due to greater uncertainty of the detection window. Attempts to overcome problems associated with poor chromatographic reproducibility by using standard flow methods [26–29] enable transfer of accurate peptide retention times between similar systems and reduces concerns about variable ion suppression effects. Thus, we implemented the workflow by using identical chromatographic gradients on Agilent 6550 QToF and Agilent 6460 QqQ mass spectrometers, instruments with highly similar ion optics and collision cells, coupled to identical 1290 UHPLC systems operating at standard flow rates (0.4 mL/min). We tested the utility of spectral libraries generated on the QToF system for methods development on the QqQ system by monitoring *y*-ions of 100 peptides that were identified from shotgun proteomic analysis of a *S*. *cerevisiae* whole cell lysate. Our results showed that the top three *y*-ions of all peptides were the same and in nearly identical order of abundance on these two systems (Panorama link: https://panoramaweb.org/rapid-shotgun-to-SRM-workflow.url).

**Fig 2:**
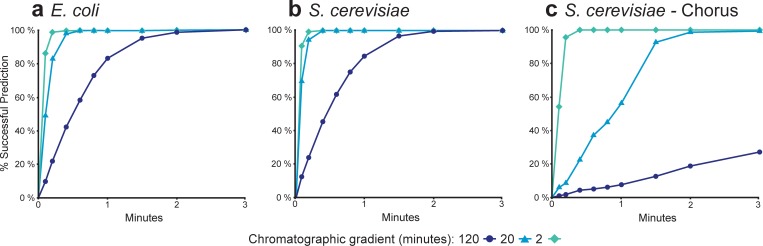
Comparison of the accuracy of peptide retention time prediction for different RT windows from proteomic libraries constructed from in-house and public MS data. The percentage of peptides that were detected of a selection of five hundred (500) peptides from (a) an in-house *E*. *coli* library; (b) an in-house *S*. *cerevisiae* library; and (c) a *S*. *cerevisiae* library downloaded from the Chorus Project for chromatographic gradients of 120 minutes (Circle), 20 minutes (Triangle), and 2 minutes (Diamond).

While direct validation of shotgun proteomic data on a QqQ mass spectrometer is important, significant value can be gained by shortening the chromatography conditions for the targeted proteomic experiments to increase sample throughput. Consequently for short chromatographic gradients, we used additional information in the spectral libraries, such as peptide retention time, ion intensity, fragmentation spectra, to develop scheduled SRM methods directly without empirically measuring peptide retention times on the new gradients. Accurate prediction of peptide retention time plays critical role in determining the number of transitions that can be measured in a single scheduled SRM run by reducing the time window required in dynamic/scheduled SRM experiments. Several algorithms have been developed to to predict peptide retention time based on their sequence information and HPLC system calibration using peptide retention standards [30]. More recently, Escher *et al*. developed iRT, an empirically-derived peptide retention time prediction method, that assigns fixed index values for peptides in relate to a set of reference peptides [23]. And, Vialas at al. showed that use of iRT prediction enhances reproducibility across different laboratories [31]. In this study, we tested our workflow with iRT prediction by directly targeting 500 peptides from *E*. *coli* and 500 peptides from *S*. *cerevisiae* for analysis using scheduled SRM methods with 120, 20, and 2 minutes chromatographic gradients. These 500 peptides and their top four product ions were chosen based on their intensity order in the library and analyzed with RT windows of six, three, and two minutes to determine the success rate of gradient transfer methods (Fig 2). All of the selected peptides from *E*. *coli* and *S*. *cerevisiae* were captured within 2 minutes RT windows for the 120 minute gradient (Fig 2A and 2B), whereas all peptides were detected for the 20 and two minute gradients within 0.5 minutes and 0.2 minutes of the predicted retention times, respectively. Overall, the measured RT of the peptides for both organisms deviated from their predicted values by less than 0.6, 0.15, and 0.1 minutes for the 120, 20, and two minute gradients, respectively (S1 Table). These much smaller retention time differences in the shorter gradient method are most likely due to the narrow peptide elution range that decreases the iRT prediction error. Similarly, we evaluated the iRT prediction accuracy in our workflow for same 500 *S*. *cerevisiae* peptides chosen from the *S*. *cerevisiae* library constructed from the raw data downloaded from Chorus [32] (Fig 2C). By using the same set of reference peptides as used in the in-house yeast proteome library we used iRT to predict RT for 120, 20, two minute chromatographic gradients. Our results showed that the RT prediction was less accurate for this library than from the in-house libraries iRT calculator in every chromatographic condition under test (S2 Table). The lower accuracy increases the effort required to implement our workflow, especially for long chromatographic gradients. The iRT prediction from in-house and external libraries is much more accurate for very short chromatographic gradients, achieving less than 0.4 minutes retention time differences of all peptides in both cases. This suggests that follow-up validation targeted proteomic experiments should be analyzed with short chromatographic gradients.

### Demonstration of the workflow by using additional biotechnology host organisms

Next, we used the standard flow UHPLC–QTOF-MS operating with DDA acquisition mode to performed standard shotgun proteomic analyses on cell lyses tryptic digests of six additional organisms without any online or offline fractionation. The organisms selected include microbes commonly used for metabolic engineering, such as *E*. *coli* and *S*. *cerevisiae* as well as emerging hosts that are attractive due to their various metabolic capabilities (Table 1). *Pseudomonas putida* is favorable for its high biomass yield, versatile metabolism, and low maintenance demand whereas *Corynebacterium glutamicum* utilizes mixed carbon sources, and has been used safely to produce various amino acids and non-natural products in food biotechnology for more than 50 years [33]. *Agrobacterium tumefaciens* is a vector for plant genetic engineering, which enables metabolic engineering of plant cells to produce high value compounds. And, *Rhodosporidium toruloides* contains multiple biotechnologically important enzymes, is capable of accumulating high percentage of lipids, and has recently been shown to effectively produce terpene compounds [34].

**Table 1:** Summary of discovery proteomic-based spectral libraries of microbes commonly used in biotechnology research and development.

Organisms	Capabilities/Utility	Unique peptides	Total proteins	Source
***Escherichia coli* DH 5α**	Model organism; Wide range of engineering tools	6994	1017	This study
***Pseudomonas putida* KT2440**	Aromatic compound degradation; Redox enzymes; Stress tolerance	1498	549	This study
***Corynebacterium glutamicum* ATCC 13032**	Amino acid production; Consumes a broad range of carbon sources	1123	358	This study
***Agrobacterium tumefaciens* EHA1**	Plant mutagenesis	1365	483	This study
***Rhodosporidium toruloides* NP11**	Lipid production; Lignin monomer utilization	1903	682	This study
***Pseudomonas putida F1***	Versatile metabolism; Aromatic compound degradation	5281	1483	[36]
***Saccharomyces cerevisiae* BY4741**	Model organism; Robustness and tolerance towards harsh fermentation conditions	32476	4184	[32]

Due to the stochastic nature of DDA acquisition methods, more proteins are added into a spectral library if the same strain were analyzed multiple times. The ability to expand the library of proteins for a given organism is very useful to metabolic engineering because a large fraction of a host proteome is absent (or very poorly expressed) in any given environment [35], thus the depth of proteome spectral library could be increased by analyzing the hosts cultured in multiple conditions. The number of unique peptides and total proteins shown in the constructed spectra library of each organism are listed in Table 1. Although standard flow LC-MS/MS is capable of capturing major proteins and producing highly reproducible proteomic data, a greater number of additional proteins were identified by traditional nano-flow LC-MS/MS approaches due to their higher sensitivity. To extend the workflow to take advantage of community resources, we also built spectral libraries from raw data acquired from public proteomic data repositories, such as data from a comprehensive proteomic analysis of *Pseudomonas putida* F1 from PRIDE (PXD001219), and data of the one-hour *S*. *cerevisiae* BY4741 proteome (Chorus Project name: SingleShot_Fusion) [32].

**Fig 3:**
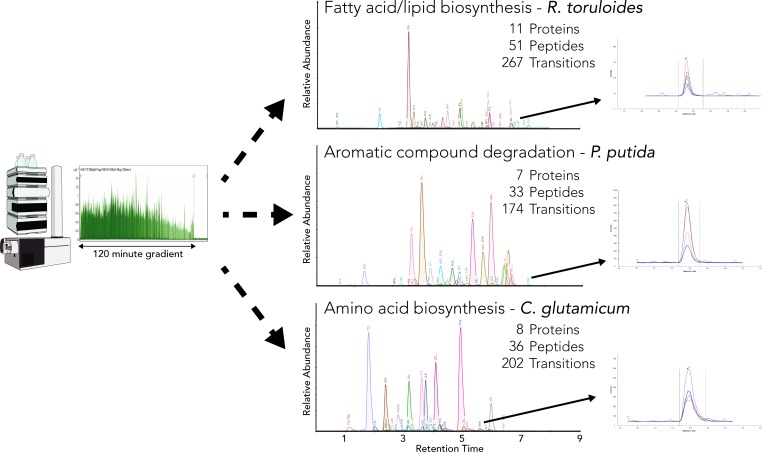
**Examples of rapid MRM target method development for quantifying pathway proteins in *R*. *toruloides* (top), *P*. *putida* (middle), and *C*. *glutamicum* (bottom).** Peptides of pathway proteins selected from spectral libraries generated via in-house shotgun proteomics or online databases.

After generating spectral libraries we applied the workflow to rapidly target proteins for several of these organisms (Fig 3). In each organism we targeted multiple proteins of interest to biotechnology research. Proteins were targeted in amino acid biosynthesis for *C*. *glutamicum*, aromatic compound degradation and L-lysine catabolism for *P*. *putida*, and fatty acid/lipid biosynthesis in *R*. *toruloides*. Target peptides were selected from the spectral libraries and refined based on size, sensitivity, the lack of modifications, and favorable tryptic digestion characteristics. The peptide identities were confirmed by the observation of at least four (most peptides had five) co-eluting y-series transitions from the precursor ion, expected y-series ion intensities from the spectral libraries, and the measured elution time was compared to the iRT prediction (S3 Table).

### Application of the workflow to test carbon source growth conditions of *P*. *putida* cultures

**Fig 4:**
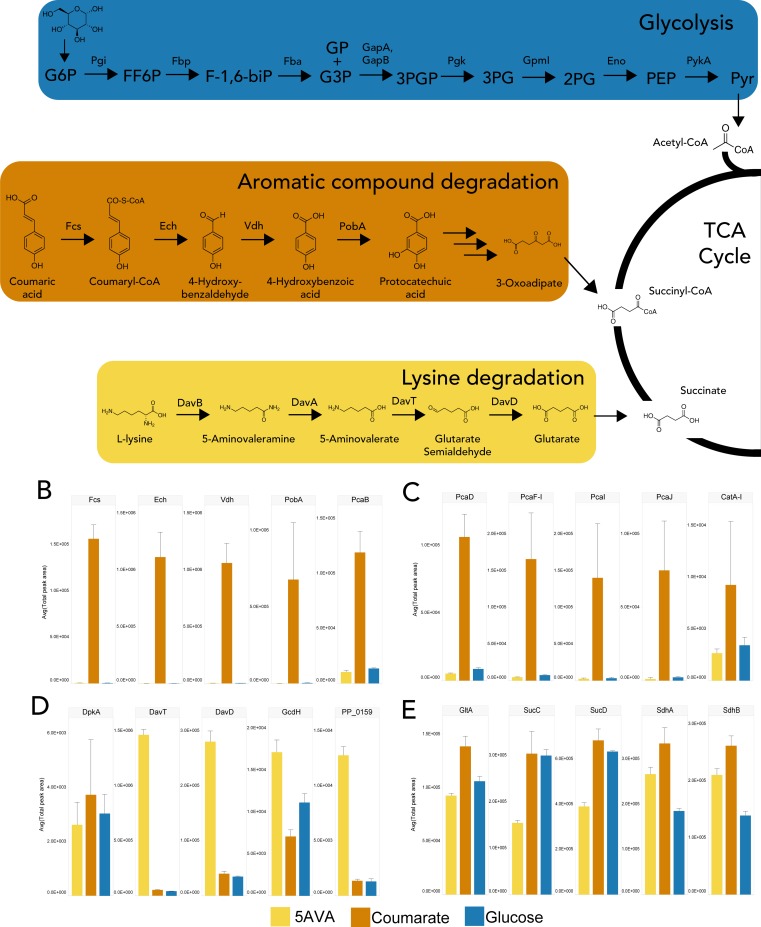
**(A) Central carbon pathways (glycolysis, lysine degradation, aromatic monomer degradation pathways, and tricarboxylic acid (TCA) cycle) in *P*. *putida*; (B-E) comparison of the relative protein abundances of *P*. *putida* grown on 10 mM of glucose, p-coumarate, and 5-aminovalerate carbon sources in MOPS media.** The error bar shows the standard deviation of measured peak area of three biological replicates. Statistical significance of p-coumarate and 5-aminovalerate against glucose were calculated by moderated t-test with the limma package in R, and resulting *p*-values were adjusted using the Benjamini-Hochberg (BH) method. *, **, and *** indicate adjusted P < 0.05, 0.01 and 0.001, respectively.

We applied this workflow to compare the proteomic profiles of carbon metabolism in *P*. *putida* when grown of three different carbon sources: glucose, *p*-coumarate, and 5-aminovalerate, an intermediate of L-lysine degradation pathway (Fig 4A). Glucose and *p*-coumarate are two of the primary components of deconstructed cellulosic biomass that is used for biofuel and bioproduct production. Coumarate is a major product of lignin hydrolysis and is metabolized by *P*. *putida* to protocatechuic acid before being brought into the TCA cycle from β-ketoadipate [37]. Whereas *P*. *putida* metabolizes L-lysine to 5-aminovalerate [38] and subsequently to glutarate which can then be brought into the TCA cycle or used to produce valuable diacids and lactams [39,40]. Interestingly, glucose metabolism by *P*. *putida* occurs primarily by the Entner-Doudoroff (ED) pathway which favors NADPH formation [41]. Here, Three biological replicates of wild-type *P*. *putida* KT2440 cells were cultured in MOPS media supplemented with 10 mM glucose, 10 mM *p*-coumarate, or 10 mM 5-aminovalerate for 16 hours and then sampled for proteomic analysis. The *P*. *putida* KT2440 spectral library was used to target proteins from the glycolysis, Pentose Phosphate pathway, tricarboxylic acid (TCA) cycle, lysine degradation, and aromatic monomer degradation pathways.

The selected iRT peptides from nine 50S and 30S ribosome proteins showed similar quantity among all tested conditions (S1 Fig) while many proteins were differentially produced based on the choice of carbon sources. For cells grown under 10 mM *p*-coumarate, the four proteins, feruloyl-CoA-synthetase (Fcs), enoyl-CoA hydratase/aldolase (Ech), vanillin dehydrogenase (Vdh), and p-hydroxybenzoate hydroxylase (PobA), involved in converting the substrate to protocatechuate, were produced in large amounts relative to their levels in the other culture conditions (Fig 4B). The genes involving the immediate conversion of protocatechuate toward TCA cycle intermediate were also produced at higher levels in the samples from coumarate-containing media (Fig 4C). Cells grown in 10 mM 5-aminovalerate yielded similar results for protein in the lysine degradation pathway. The 5-aminovalerate aminotransferase (DavT) and glutarate-semialdehyde dehydrogenase (DavD) were highly expressed in these cells to convert the substrate to glutarate, which can be fed into TCA cycle (Fig 4D). We also observed that Glutaryl-CoA dehydrogenase (GcdH) and PP_0159, a putative CoA transferase, were highly expressed in these cells (Fig 4D). Their elevated levels could be a response of metabolite flow from glutarate to glutaryl-CoA and other steps further down the pathway toward central carbon metabolism. Since the metabolite flow of *p*-coumarate and 5-aminovalerate are towards TCA cycle intermediates, we expected the glycolysis and pentose phosphate pathway proteins to be similar between these two sample groups (S1 Fig). Indeed, we observed that glyceraldehyde-3-phosphate dehydrogenase (GapA), Pyruvate dehydrogenase E1 component (AceE), and glucose-6-phosphate 1-dehydrogenase (ZwfA) in these two pathways are the most noticeable protein quantity differences among the three sample groups, and cells grown under 5-aminovalerate and coumarate had similar but lower levels of these proteins than cells grown under glucose. Among the TCA cycle proteins, we observed slightly higher amounts of citrate synthase (GltA), a regulated TCA cycle protein, in samples from coumarate-containing media (Fig 4E). A lower expression of succinate-coA synthase subunits (SucC and SucD) was observed in 5-aminovalerate samples in comparison to glucose and coumarate samples. On the other hand, the succinate dehydrogenase subunits (SdhA and SdhB) were expressed higher in both 5-aminovalerate and coumarate samples than glucose samples (Fig 4E). Both of these observations can be explained by an abundance of succinate, the end product of the recently described glucogenic route of glutarate catabolism, an intermediate of 5-aminovalerate metabolism [39].

## Discussion

Quantitative proteomic studies play an important role in assessing how an organism changes under different environmental, stress, or engineering conditions. Thus, the process of selecting high-quality, quantitative peptides for targeted proteomic experiments is typically lengthy and involved. Our work details a workflow that couples accurate, reproducible chromatography with the information in proteomic spectral libraries to enable the translation of data from shotgun proteomic experiments to high-throughput targeted proteomic methods. The workflow described here offers a rapid means to validate a large number of peptides that may be false positive identifications, thus providing a powerful method to increase confidence in many peptides from large DDA/DIA datasets. Resources such as the human SRMAtlas [12], implementation of retention time standards, and the Biodiversity Library [19] complement this workflow and greatly aid development of targeted proteomic methods from large datasets. While this workflow does not remove the need to screen for chemical or biological interferences that could hinder quantitative analysis, it does greatly reduce the amount of time and effort necessary to target peptides of interest from shotgun proteomic analyses, optimize short chromatographic methods, and transfer methods between different types of mass spectrometers.

When applying this workflow care must be taken to eliminate possible interferences such as co-elution of peptides from background/matrix proteins especially for very short gradient acquisitions and for eukaryote proteomic research where isoforms and post-translational complicate peptide validation. Capturing quantitative proteomic information for many conditions will enable construction of detailed, accurate metabolic models [42] that predict phenotypic responses for both basic and applied scientific goals. Targeted proteomic assays, in particular, have grown into key components of these types of studies because of their flexibility, specificity, and sensitivity. By using the workflow described here, peptides from large online repositories of shotgun proteomic experiments can be rapidly optimized for targeted proteomic data acquisition. Reducing the time and effort to test interesting proteins from shotgun proteomic experiments encourages secondary validation of these data, thus providing a powerful method to increase confidence in many proteomic studies.

Overall, this workflow can be applied to a broad set of proteomic analyses with minimal development time. It is enabled by highly reproducible peptide retention times from standard-flow chromatography systems, comprehensive spectral libraries produced from shotgun proteomic experiments, and it offers a rapid means to validate peptides that may be false positive identifications. These characteristics aid broad, multi-lab projects by improving repeatability and reproducibility across different systems and facilitate data comparisons beyond what one lab can achieve. As LC-MS systems continue to improve and the proteomic community contributes to data repositories, this workflow will help lower the barrier to realizing the full potential of proteomics in medical and biotechnology research.

## Supporting information

S1 Table Peptide retention time prediction based on spectra library built with in house MS data. (XLSX)

S2 Table Peptide retention time prediction based on spectra library built with public depository MS data. (XLSX)

S3 Table Peptide retention time prediction of industrial interested pathway proteins from various in house built spectra libraries. (XLSX)

S1 Fig The relative abundances of Glycolysis, Pentose phosphate pathway, TCA cycle, and Ribosome proteins in *P*. *putida* grown on 10 mM of glucose, p-coumarate, and 5-aminovalerate carbon sources in MOPS media. The error bar shows the standard deviation of measured peak area of three biological replicates. Statistical significance of p-coumarate and 5-aminovalerate against glucose were calculated by moderated t-test with the limma package in R, and resulting *p*-values were adjusted using the Benjamini-Hochberg (BH) method. *, **, and *** indicate adjusted P < 0.05, 0.01 and 0.001, respectively.
(PDF)
