# Supplementary figures and images for: A rapid methods development workflow for high-throughput quantitative proteomic applications
Source: PLoS One. 2019 Feb 14;14(2):e0211582. doi: 10.1371/journal.pone.0211582 (PMC6375547; doi:10.1371/journal.pone.0211582)

# TCA cycle proteins

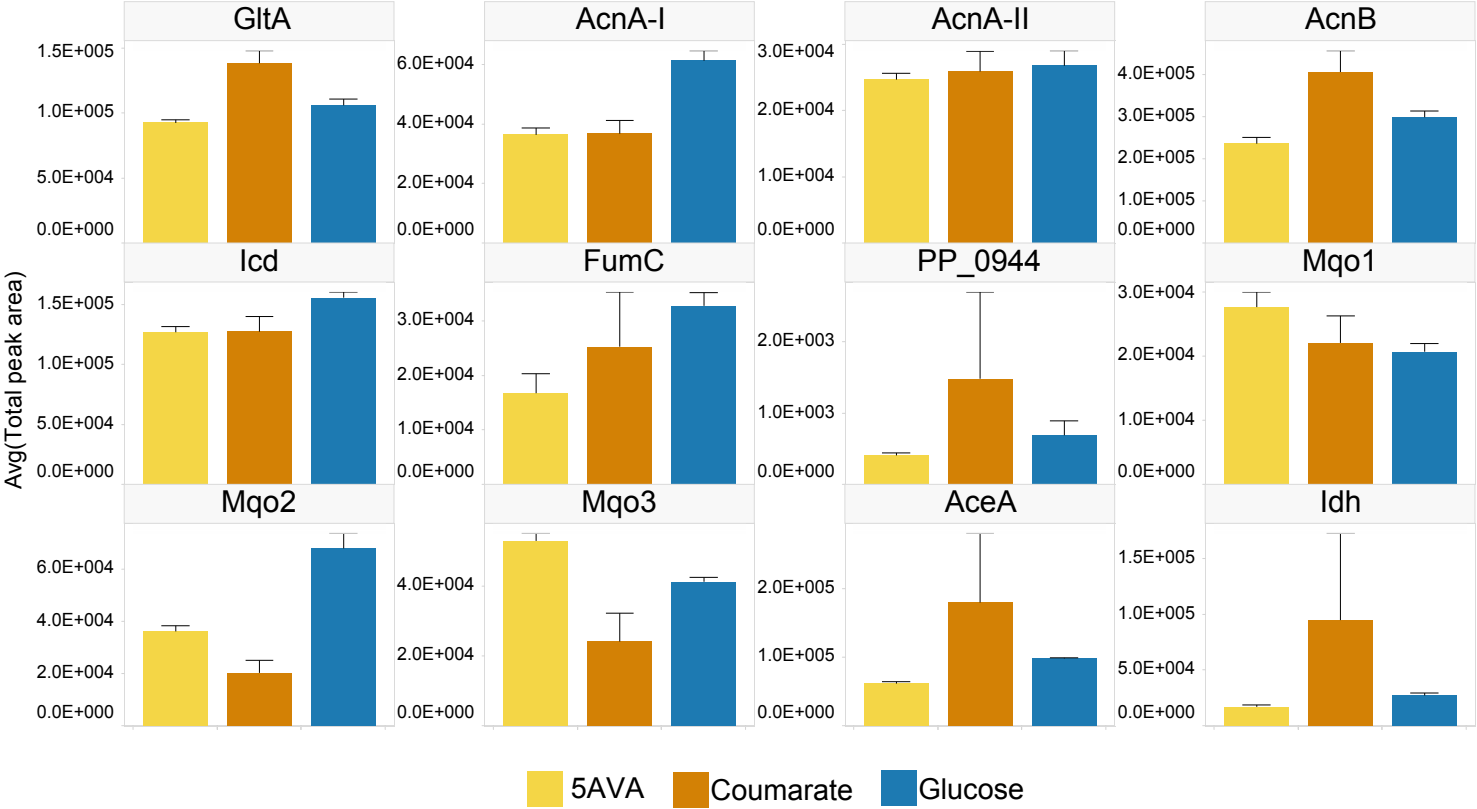

# Glycolysis pathway proteins

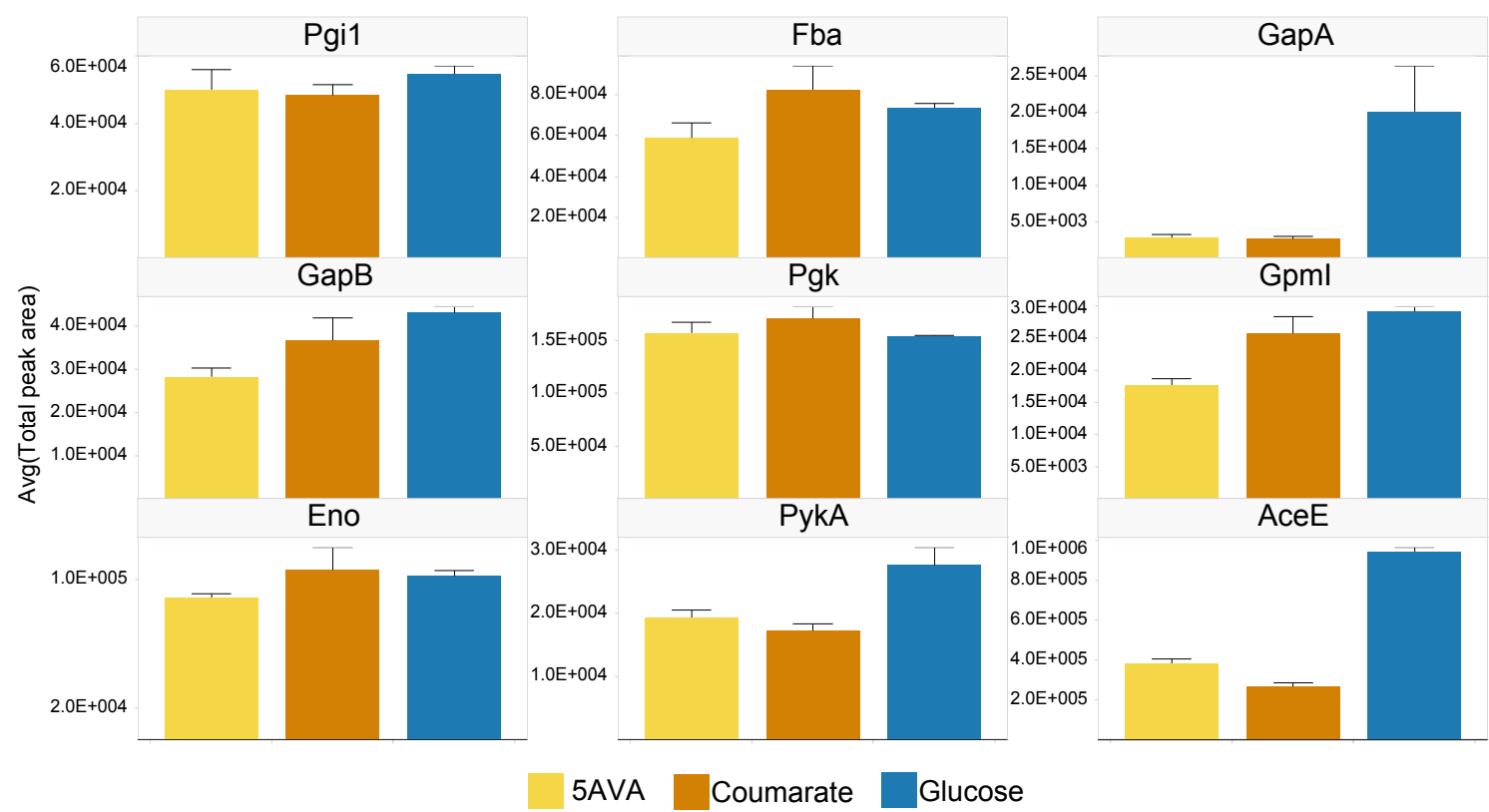

# Pentose phosphate pathway proteins

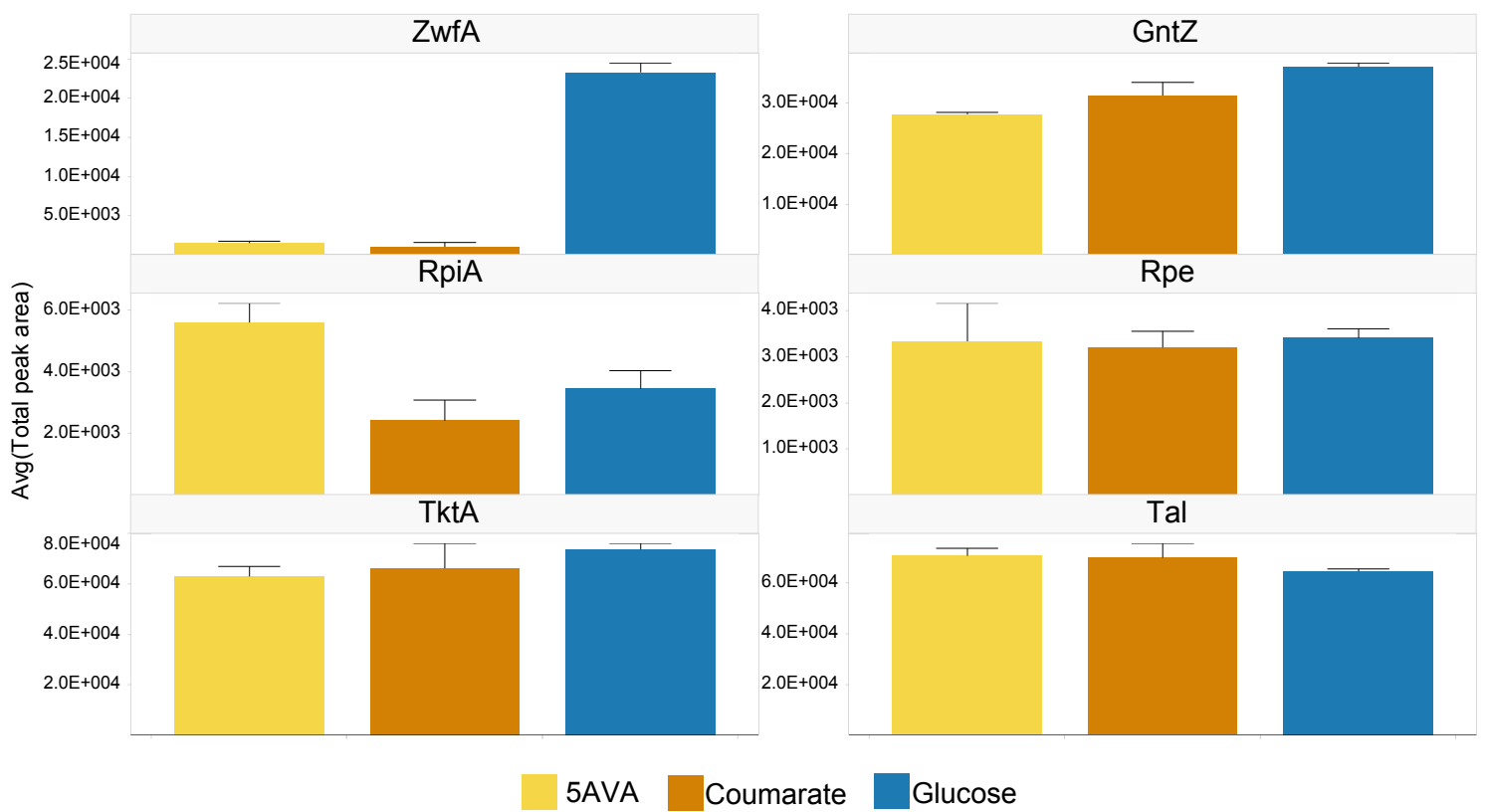

Supplement: S1 Fig — The error bar shows the standard deviation of measured peak area of three biological replicates. Statistical significance of p-coumarate and 5-aminovalerate against glucose were calculated by moderated t-test with the limma package in R, and resulting p-values were adjusted using the Benjamini-Hochberg (BH) method. *, **, and *** indicate adjusted P < 0.05, 0.01 and 0.001, respectively. (PDF) [file pone.0211582.s004.pdf]
